# MicroRNA and Transcription Factor Mediated Regulatory Network Analysis Reveals Critical Regulators and Regulatory Modules in Myocardial Infarction

**DOI:** 10.1371/journal.pone.0135339

**Published:** 2015-08-10

**Authors:** Guangde Zhang, Hongbo Shi, Lin Wang, Meng Zhou, Zhenzhen Wang, Xiaoxia Liu, Liang Cheng, Weimin Li, Xueqi Li

**Affiliations:** 1 Department of Cardiology, The Fourth Affiliated Hospital of Harbin Medical University, Harbin, Heilongjiang 150001, PR China; 2 College of Bioinformatics Science and Technology, Harbin Medical University, Harbin, Heilongjiang 150081, PR China; 3 Department of Cardiology, The First Affiliated Hospital of Harbin Medical University, Harbin, Heilongjiang 150001, PR China; University of Pittsburgh, UNITED STATES

## Abstract

Myocardial infarction (MI) is a severe coronary artery disease and a leading cause of mortality and morbidity worldwide. However, the molecular mechanisms of MI have yet to be fully elucidated. In this study, we compiled MI-related genes, MI-related microRNAs (miRNAs) and known human transcription factors (TFs), and we then identified 1,232 feed-forward loops (FFLs) among these miRNAs, TFs and their co-regulated target genes through integrating target prediction. By merging these FFLs, the first miRNA and TF mediated regulatory network for MI was constructed, from which four regulators (SP1, ESR1, miR-21-5p and miR-155-5p) and three regulatory modules that might play crucial roles in MI were then identified. Furthermore, based on the miRNA and TF mediated regulatory network and literature survey, we proposed a pathway model for miR-21-5p, the miR-29 family and SP1 to demonstrate their potential co-regulatory mechanisms in cardiac fibrosis, apoptosis and angiogenesis. The majority of the regulatory relations in the model were confirmed by previous studies, which demonstrated the reliability and validity of this miRNA and TF mediated regulatory network. Our study will aid in deciphering the complex regulatory mechanisms involved in MI and provide putative therapeutic targets for MI.

## Introduction

Myocardial infarction (MI), defined as myocardial cell death due to prolonged myocardial ischemia, is a leading cause of mortality and morbidity worldwide [1]. Notably, acute MI accounts for most of the mortality associated with coronary artery disease. Indeed, according to a report from the American Heart Association, approximately every 34 seconds, one American has a coronary event, and approximately every 1 minute 24 seconds, an American will die from this event [1]. To date, however, the molecular mechanisms underlying MI are still not fully understood.

Gene regulatory networks modulate the entire process of gene expression and protein formation in living cells, and therefore determine the fate of cells. MicroRNAs (miRNAs) and transcription factors (TFs) are the main regulators of these networks and thus participate in the regulation of many important biological processes, including cell proliferation, differentiation and apoptosis. Naturally, the dysregulation of miRNAs and TFs is associated with a broad range of diseases, including MI. Therefore, understanding the miRNA and TF mediated regulatory network of MI will shed light on the mechanisms of it pathogenesis.

MiRNAs are endogenous, small non-coding RNAs (~22nt) that inhibit gene expression by binding to the 3’ untranslated regions (3’ UTRs) of target mRNAs [2]. They regulate gene expression at the posttranscriptional level. A growing body of evidence has demonstrated the crucial roles of miRNA in MI and many other human diseases [3, 4]. Indeed, elevated levels of miR-1 and miR-133a in the serum of patients with cardiovascular disease was a reported indication of myocardial damage [5]. In murine cardiomyocytes, miR-150 was found to protect the mouse heart from ischemic injury by regulating cell death [6]. Additionally, miR-34a was reported to regulate cardiac fibrosis after myocardial infarction through the targeting of Smad4 expression [7].

TFs are regulators of gene transcription at the transcriptional level, albeit as modular proteins that bind to DNA-binding domains in the promoter region of target genes [8]. Regulation of both miRNAs and TFs is tightly linked, and they share similar regulatory logics [9–11]. Moreover, they act in a largely combinatorial manner, cooperatively regulating the same target genes. As miRNAs and TFs may also mutually regulate one another, feed-forward loops (FFLs) comprising miRNAs, TFs and genes thus exist [11]. Gene regulatory network analysis has demonstrated that FFLs comprise recurrent network motifs in the mammalian regulatory network [12, 13]. Therefore, deciphering the involvement of FFLs in the pathogenesis of complex human diseases will provide new clues for understanding specific biological events. Currently, revealing molecular mechanisms underlying complex diseases based on FFLs has already produced valuable results [14–17]. For example, Ye et al. found that miR-19 inhibited CYLD in T-cell acute lymphoblastic leukemia using identified FFLs [14]. Sun et al. extended 3-node FFLs to 4-node FFLs and constructed the first miRNA-TF regulatory network for glioblastoma [15]. In addition, Yan et al. and Peng et al. proposed different computational methods for identifying FFLs in human cancers using parallel mRNA and miRNA expression profiles [18, 19].

In this study, we constructed the first miRNA and TF mediated regulatory network for MI based on three specific types of FFLs. We then systematically analyzed the global properties of this network and identified several important regulators and regulatory modules within the network. Additionally, based on network analysis and a comprehensive literature review, we proposed a pathway model demonstrating the potential co-regulation of miR-21-5p, the miR-29 family and SP1 during MI.

## Materials and Methods

### Collection of genes and miRNAs related to MI

DisGeNET [20] is a new human gene-disease database integrating several widely used human gene-disease databases, such as the Online Mendelian Inheritance in Man (OMIM) database [21], the Genetic Association Database (GAD) [22], the Mouse Genome Database (MGD) [23], the Comparative Toxicogenomics Database (CTD) [24], PubMed and Uniprot [25]. In this study, 854 unique MI-related genes (MIgenes) were selected from the DisGeNET (May 2014) database.

MI-related miRNAs (MImiRNAs) were selected by performing a comprehensive literature review. First, a group of relevant articles were compiled from three manually curated and experimentally verified human disease-miRNA association databases: miR2Disease [26], HMDD (version 2.0) [27] and PhenomiR (Feb 2011) [27] using the search phrase “myocardial infarction” and from PubMed using the search phrase “myocardial infarction AND microRNA”. Each article was then manually searched for miRNAs with dysregulated expression in MI. These miRNAs were then mapped to mature miRNAs based on the database of human miRNAs from miRBase (release 21) [28], and 78 unique mature MImiRNAs were ultimately selected.

### Identification of miRNA-gene/TF regulatory relationships

MiRNA-gene regulatory relationships were assessed using both experimentally verified and predicted targets of the 78 selected MImiRNAs. Experimentally verified targets were obtained from TarBase (version 6.0) [29], miRTarBase (version 4.5) [30] and miRecords (version 4) [30] databases, and predicted targets were obtained from TargetScan (version 6.2) [31], miRDB (version 5.0) [31] and TargetMiner (May 2012) [32] databases. To increase the reliability of the results, only the targets appearing in at least two databases were retained in this study.

To acquire regulatory relationships of miRNA-TF, a gene list of 1698 unique human TFs from Transfac (April 2012) [33], TRED [34], TransmiR (version 1.2) [35] and a previously defined TFs in a previous report [36] were extracted. These genes were regarded as TFs. We implemented the above procedure and obtained the relationships between miRNAs and TFs.

### Identification of TF-gene/miRNA regulatory relationships

TF-gene regulatory relationships were integrated using experimentally verified and predicted TF targets obtained from UCSC, TRED [34] and Transfac (April 2012) [33] databases. Experimentally verified TF target genes were retrieved from TRED [34] and Transfac [33] databases. Predicted targets were obtained from two files (TFbsConFactors.txt and TFbsConsSites.txt) containing the predicted transcription factor binding site (TFBS) information downloaded from UCSC hg19, and TFBSs were made to be conserved among humans, mouse and rats. To further reduce false positive predictions of TFBSs, a Z score of 2.33 was selected as a cut-off. A TFBS was considered to be associated with a target gene when it was in the promoter region of the gene and its Z score was larger than 2.33. The promoter region of a gene was defined as a 1-kb region up- and down-stream of the transcription start site, according to the ENCODE project [37].

Experimentally verified TF-miRNA regulatory relations were obtained from TransmiR (version 1.2) [35], and predicted TF miRNA targets using UCSC. MiRNA precursor sequences were obtained from the miRBase (release 21) [28] database, and 2-kb upstream of pre-miRNAs were selected as their putative promoter regions. Similar to the process of predicting TF-gene regulatory relationships, the predicted TF-miRNA relations were obtained.

### Randomization test of FFLs

**Fig 1:**
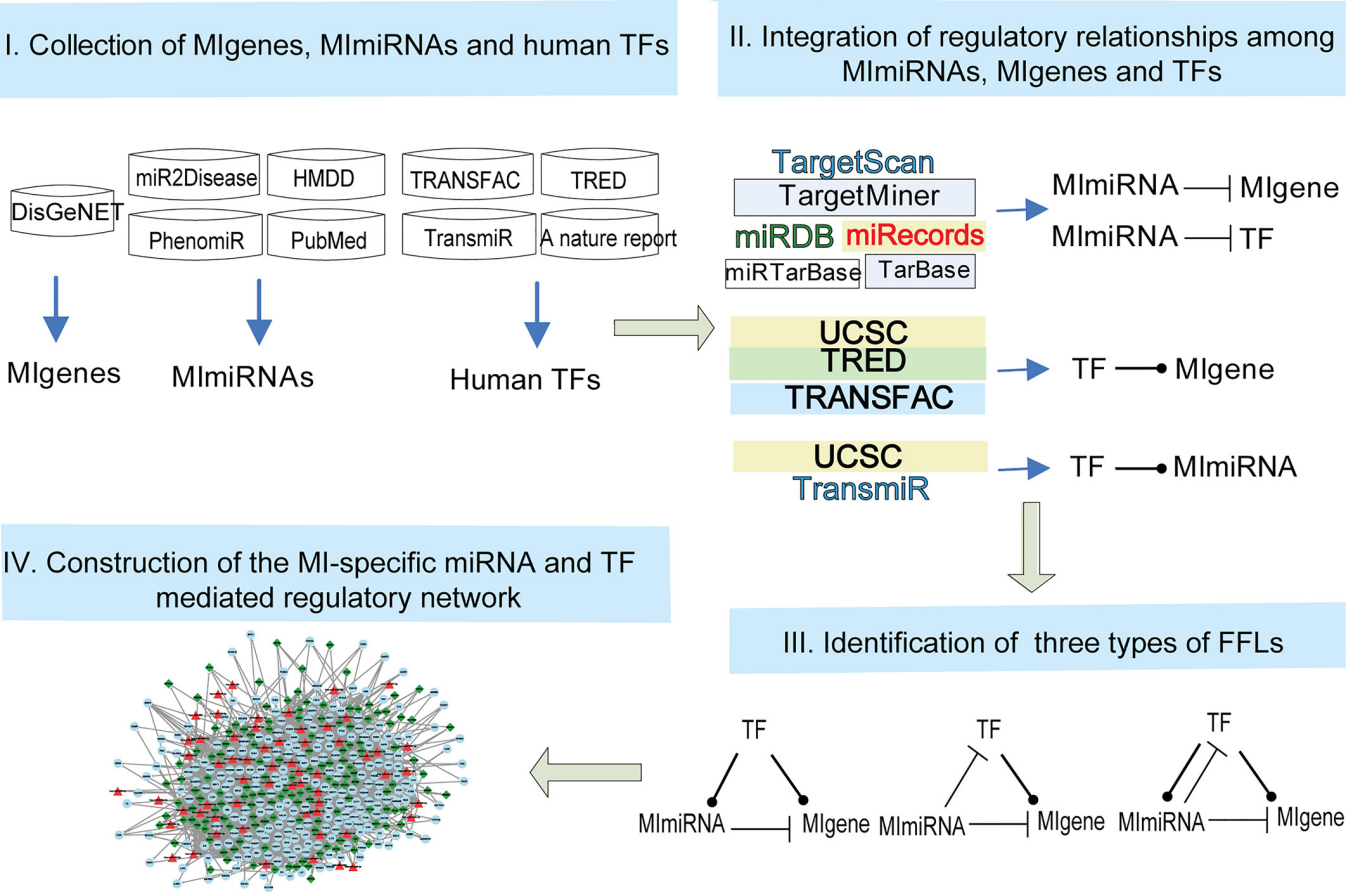
Workflow to construct the MI-specific miRNA and TF mediated regulatory network. Step 1: Collecting MIgenes, MImiRNAs and known human TFs from publicly available databases and literature. Step 2: Retrieving regulatory relationships among MIgenes, MImiRNAs and known human TFs using an integrated strategy. Step 3: Identifying three types of FFLs based on the relationships among MIgenes, MImiRNAs and known human TFs. Step 4: Constructing the MI-specific miRNA and TF mediated regulatory network by merging the FFLs obtained in step 3.

Randomization test was performed to evaluate the significance of the FFLs observed in the set of TFs, MImiRNAs and MIgenes (Fig 1) according to a previous study [13]. In each run of the randomization, the same number of miRNA-gene pairs from all MImiRNA target genes was randomly selected, and the number of FFLs was then calculated. This procedure was implemented 10,000 times, and a p-value was calculated as the proportion of randomly achieved FFLs greater than or equal to the number of true FFLs.

### Identification of network modules

The clique percolation clustering method was used to identify network modules [38]. A clique in the miRNA and TF mediated regulatory network was a complete subgraph with every two nodes linked by an edge. A module, otherwise known as a k-clique community, was obtained by merging all k-cliques (a clique wherein the number of nodes in a complete subgraph was k), and these k-cliques could be connected to each other through adjacent k-cliques with k-1 common nodes. CFinder software [39] was used to identify tightly connected network modules.

### Computation of Gene Ontology semantic similarity

Gene Ontology (GO) semantic similarity scores based on GO terms for each pair of genes were computed using the R GOSemSim package [40]. For each of the three GO sub-ontologies (biological process, molecular function and cellular component), the semantic similarity scores were calculated for all gene pairs in a module. To examine the significance of the functional similarity of genes in a module, a randomization test was performed. For a given module, the same number of genes in the module were selected from the 854 MIgenes, and their GO semantic similarities were analyzed. This procedure was performed 1,000 times, and a Kolmogorov-Smirnov test (KS-test) was used to assess whether the GO semantic similarity scores of all gene pairs from the module were significantly higher than that of randomly selected pairs.

## Results

### Regulatory relationships among genes, miRNAs and TFs

The regulatory relationships among genes, miRNAs and TFs (Fig 1) were limited to 854 MIgenes, 78 MImiRNAs and 1,698 known human TFs. The results are shown in Table 1.

**Table 1:** Summary of regulation relationships among MIgenes, MImiRNAs and TFs.

Relationship	No. of pairs	No. of miRNAs	No. of genes	No. of TFs
miRNA-gene^a^	1444	74	447	-
miRNA-TF^b^	3322	76	-	862
TF-gene^c^	4369	-	651	462
TF-miRNA^d^	214	48	-	116

^a^miRNA repression of gene expression.

^b^miRNA repression of TF expression.

^c^TF regulation of gene expression.

^d^TF regulation of miRNA expression

#### miRNA-gene

In total, 1,444 miRNA-gene pairs, including 74 MImiRNAs and 447 MIgenes, were obtained from experimentally verified and predicted miRNA target databases. The miRNA let-7b-5p had the largest number of target genes (84 genes).

To assess whether MImiRNAs have more targets in 854 MIgenes than in randomly selected 854 genes, a permutation was performed. For each MImiRNA, we randomly selected 854 genes from human protein-coding genes and counted the number of target genes. This randomness analysis was implemented 10,000 times and one sample t-test was used to examine the significance. Ultimately, all MImiRNAs targeted a significantly larger number of genes in MIgenes than randomly selected genes (*p*-value< 2.20×10^−16^).

#### miRNA-TF

Using the same miRNA target prediction method, among 1,698 human TFs, 76 MImiRNAs were verified or predicted to be targets of 862 TFs, forming 3,322 miRNA-TF pairs. Among the 76 MImiRNAs and 862 TFs, the miRNA miR-93-5p targeted the largest number of TFs (172 TFs), and the TF NFAT5 (nuclear factor of activated T-cells 5, tonicity-responsive) was targeted by the largest number of miRNAs (27 miRNAs). Notably, NFAT was recently found to be associated with myocardial damage and remodelling [41].

#### TF-gene

Among 854 MIgenes and 1,698 human TFs, 651 genes were verified or predicted to be targets of 462 TFs, forming 4,369 unique TF-gene pairs. Among the 462 TFs, the TF SP1 (Sp1 transcription factor; also an MIgene) targeted the largest number of genes (174 genes).

#### TF-miRNA

Among 78 MImiRNAs and 1,698 human TFs, 48 MImiRNAs were verified or predicted to be targets of 116 TFs, forming 214 unique TF-miRNA pairs. Among the 48 MImiRNAs and 116 TFs, the miRNA miR-21-5p was targeted by the largest number of TFs (18 TFs), and the TFs EGR1 (early growth response 1; also an MIgene) and MYC (v-myc avian myelocytomatosis viral oncogene homolog) both targeted the largest number of miRNAs (9 miRNAs). MYC played important roles in enhancing cardiovascular repair capacity after acute MI by interacting with other molecules [42].

### Presence and significance of feed-forward loops in MI

FFLs are motifs known to play important roles in gene regulation [12, 43]. Typically, FFLs can be classified into three types according to the main regulator [11, 44]: TF-FFL, miRNA-FFL and composite FFL (S1 Fig). In a TF-FFL, TF is the main regulator, which regulates a miRNA and their common target gene while in a miRNA-FFL, miRNA is the main regulator. In a composite-FFL, TF regulates a miRNA and a target gene, while the miRNA regulates the TF and the target gene. By combining the relationships among MImiRNAs, MIgenes and known human TFs (Table 1), we identified 1,232 FFLs, which included 236 TF-FFLs (19.16%), 902 miRNA-FFLs (73.21%) and 94 composite FFLs (7.63%). Merging the FFLs reduced the totals to 60 miRNAs, 256 genes and 141 TFs. The number of nodes and links in the FFLs is shown in Table 2 and S1 Table.

**Table 2:** Summary of three types of feed-forward loops based on MI-related data.

		Number of nodes				Number of links				
Motif	Number of FFLs	Genes	miRNAs	TFs	Total	miRNA-gene	miRNA-TF	TF-gene	TF-miRNA	Total
TF-FFL	236	109	29	33	171	191	-	184	65	440
miRNA-FFL	902	227	55	129	411	530	358	594	-	1482
Composite-FFL	94	50	9	13	72	62	18	85	18	183
Total	1232	256	60	141	457	621	376	786	83	1866

To examine whether the identified FFLs were enriched in MIgenes, 10,000 random simulations were run for each FFL type. As 1,444 MImiRNA-MIgene target pairs were originally identified, for each run of the simulation, 1,444 miRNA-gene pairs were randomly selected from all the targets of the 78 MImiRNAs, and the number of corresponding FFLs was computed. As a result, *p*-value = 0 were obtained for TF-FFL, miRNA-FFL and composite FFL separately (see Materials and Methods), which demonstrated that these FFLs were not randomly generated and indicated specific biological significance.

### Construction and analysis of the miRNA and TF mediated regulatory network in MI

**Fig 2:**
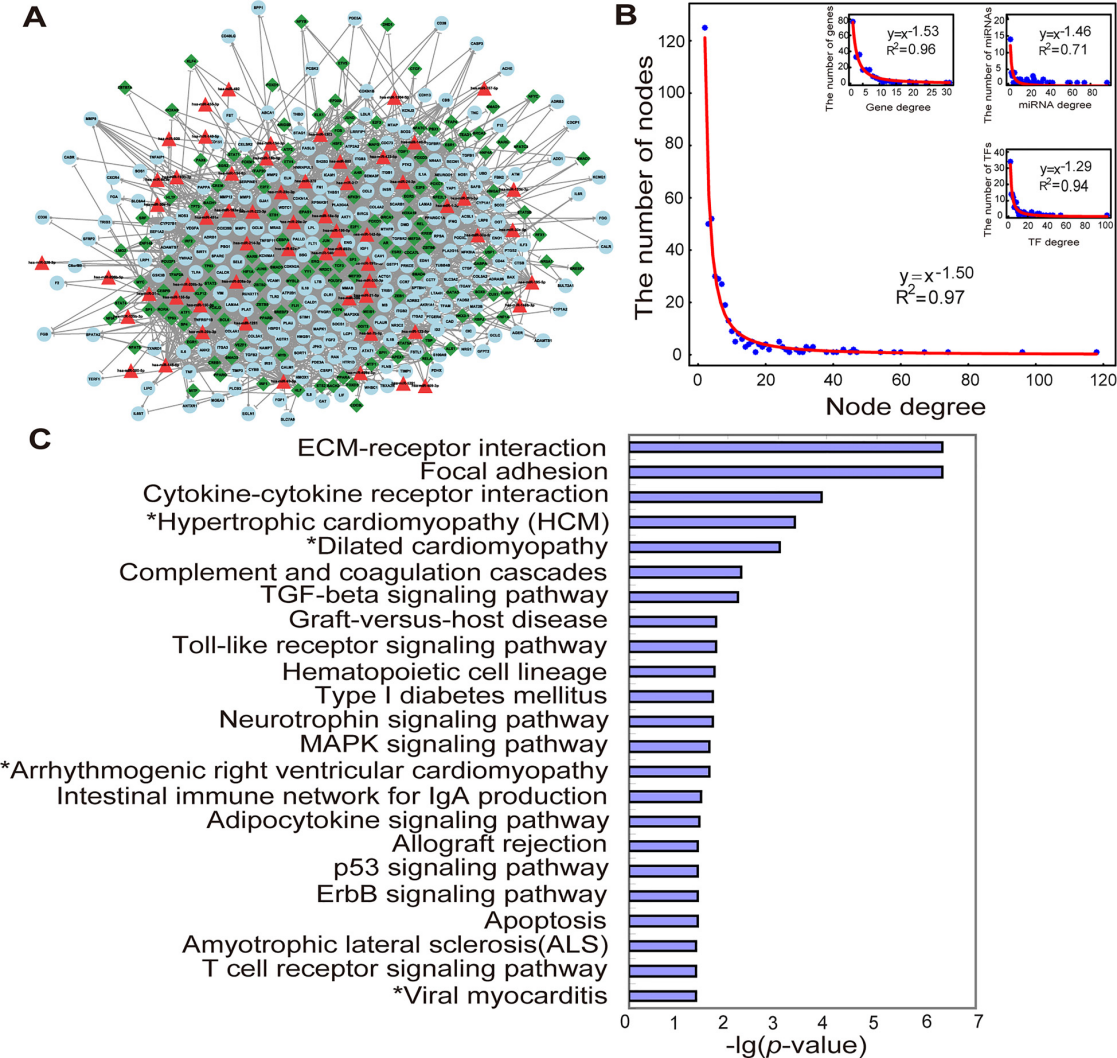
MI-specific miRNA and TF mediated regulatory network and its structure and functional features. (A). The MI-specific miRNA and TF mediated regulatory network was composed of MIgenes (circles), MImiRNAs (triangles) and TFs (diamonds). This network consists of 438 nodes and 1,780 links. (B). Degree distribution of all the nodes in the network, and degree distribution of genes, miRNAs and TFs in the network. (C). Significantly-enriched KEGG pathways for MIgenes in the network (cancer pathways removed). *p*-value was adjusted using the Benjamini-Hochberg multiple testing correction and a *p*-value of <0.05 was used as a threshold to select significant KEGG pathways. ‘*’ indicated the pathways belonged to cardiovascular disease pathways in KEGG.

We constructed a miRNA and TF mediated regulatory network specific for MI by merging the three types of FFLs identified in the above subsection (Fig 2A). The network included 438 unique nodes (60 MImiRNAs, 256 MIgenes and 141 TFs). Among 256 MIgenes and 141 TFs, 19 common genes (*APEX1*, *BRCA1*, *CREB1*, *CREM*, *ESR1*, *ESR2*, *FOS*, *FOXO3*, *HIF1A*, *MEF2A*, *NFκB1*, *NFYC*, *NR3C1*, *PPARA*, *PPARG*, *SP1*, *SREBF2*, *STAT3* and *TP53*) were observed. To more clearly analyse the features of the regulators separated from the other genes, we considered these genes only in the TF set and referred to them as MITFs. Thus, there were 438 unique nodes (60 MImiRNAs, 237 MIgenes and 141 MITFs) and 1,780 interactions in the network (S2 Table). Among the 1,780 interactions, 529 belonged to miRNA-gene pairs, 382 belonged to miRNA-TF pairs, 680 belonged to TF-gene pairs, 83 belonged to TF-miRNA pairs and 106 belonged to TF-TF pairs.

First, the global properties of this network were assessed based on network topological analyses. As shown in Fig 2B, the degree of most nodes was low, and only a few nodes interacted with a relatively large number of other nodes. The degree distribution indicated a power law with a slope of -1.50 and an R^2^ of 0.97, meaning that the network was scale-free. Notably, the individual degree distribution of genes, miRNAs and TFs was also scale-free (Fig 2B). The average node degree of genes, miRNAs and TFs was 5.10 (range 2–31), 16.57 (range 1–88) and 9.62 (range 2–102), respectively.

Next, nodes with high betweenness centrality and highly connected features (hubs), which together demonstrate that the nodes play key roles in maintaining the overall connectivity of the network, were analyzed. MiRNAs and TFs with the highest (top 5%) betweenness centrality were as follows: 3 miRNAs (miR-21-5p, miR-155-5p and miR-92a-3p) and 7 TFs (ESR1, SP1, NFκB1, TP53, MYC, STAT3 and FOXO3) (S3 Table). Betweenness centrality of all the genes in the network was zero, suggesting that these genes might displayed less powerful ability for transferring biological information compared with miRNAs and TFs. Using a method previously proposed by Yu et al. [45], 5 hub genes (*CDKN1A*, *VEGFA*, *IGF1*, *PSG1* and *TNF*), 7 hub miRNAs (miR-155-5p, let-7b-5p, miR-92a-3p, miR-93-5p, miR-21-5p, miR-29b-3p and miR-29a-3p) and 9 hub TFs (SP1, JUN, MYC, NFκB1, ESR1, NR3C1, CREB1, CEBPA and ETS1) were identified (S4 Table). Among the 9 hub TFs, JUN, MYC, CEBPA and ETS1 were newly-identified MI-related TFs; the remaining hub TFs were already classified as MIgenes.

Significantly-enriched biological pathways for 237 MIgenes in the network were also examined. Using KEGG pathway enrichment analysis of 237 MIgenes, and by applying the Benjamini-Hochberg multiple testing correction, 30 significantly-enriched pathways were identified with an adjusted *p*-value of <0.05 (S5 Table). To more clearly demonstrate these results, cancer pathways were removed (Fig 2C). Of the remaining significantly-enriched pathways, four pathways in cardiovascular diseases pathways were all significantly enriched: hypertrophic cardiomyopathy (adjusted *p*-value = 4.57×10^−4^), dilated cardiomyopathy (adjusted *p*-value = 8.81×10^−4^), arrhythmogenic right ventricular cardiomyopathy (adjusted *p*-value = 0.0247), and viral myocarditis (adjusted *p*-value = 0.0452). Several other pathways were well-known and important in MI, such as the TGF-β signalling pathway, the toll-like receptor signalling pathway, the MAPK signalling pathway and apoptosis.

### Network modules in MI

To identify network modules that may play important roles in the molecular pathology of MI, CFinder software [39] was used. Modules could only be obtained when k = 3, 4 or 5. As described in the Materials and Methods, a module is composed of adjacent k-cliques, so a large number of less tightly connected modules will be obtained at a small k-value, while increasing the k-value will generate fewer and more tightly connected modules. Thus, a k-value of 5 was chosen, and three modules were identified.

**Fig 3:**
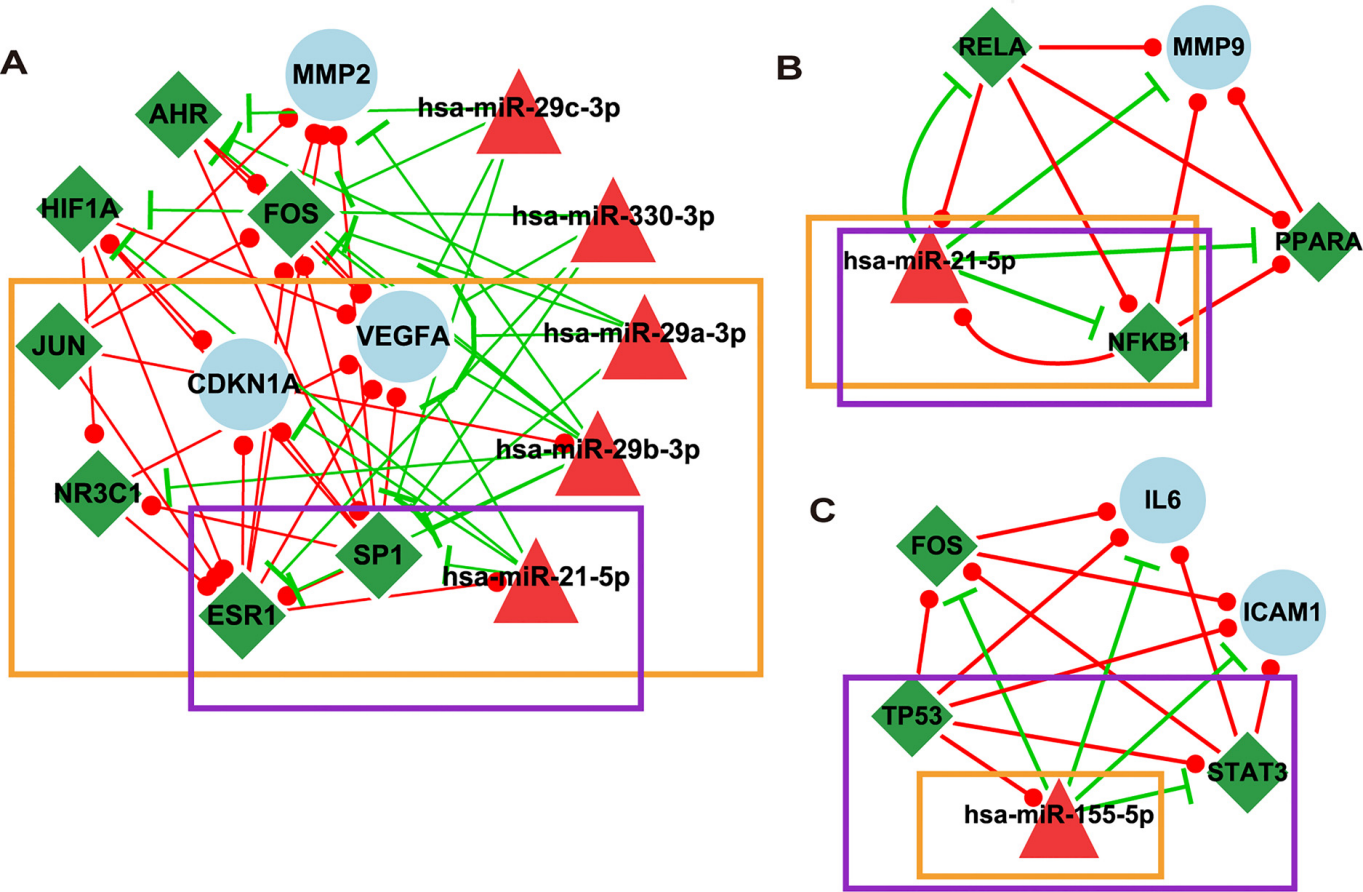
Three modules in the MI-specific miRNA and TF mediated network. The nodes in the orange box denote hub nodes, and the nodes in the purple box denote having a top 5% betweenness centrality in the network.

As shown in Fig 3A, the first module contained 15 nodes (3 MIgenes, 5 MImiRNAs and 7 MITFs). Among the 15 nodes, 9 (60.00%) were hub nodes, and 3 (20.00%) were ranked in the top 5% of 433 nodes in the network for betweenness centrality. Three (miR-29a-3p, miR-29b-3p and miR-29c-3p) of the 5 MImiRNAs belonged to the miR-29 family, members of which are known to target a cadre of protein-coding mRNAs involved in fibrosis and play crucial roles in cardiac fibrosis [46]. Further examination was performed to determine whether the genes in this module have more similar function than randomly selected MIgenes. GO semantic similarity scores based on the three sub-ontologies (biological process, molecular function and cellular component) were calculated among genes using the R GOSemSim package [40] (see Materials and Methods). The results indicated that the gene pairs in this module tended to have significantly higher semantic similarity scores than those of randomly selected MIgene pairs (S2 Fig). In addition, we investigated the important role of this module in MI-specific miRNA and TF mediated network. The module was removed and the connectivity of the network was tested. As a result, closeness centrality was significantly lower than that of original network (*p*-value = 8.32×10^−7^, t-test), which demonstrated the importance of the community in communicating information with other molecules in the network.

As shown in Fig 3B and 3C, the second module contained 5 nodes (1 MIgenes, 1 MImiRNAs and 3 MITFs). Among the 5 nodes, NFκB1 and miR-21-5p, which were hub nodes, were also ranked in the top 5% of 433 nodes in the network for betweenness centrality. The third module contained 6 nodes (2 MIgenes, 1 MImiRNAs and 3 MITFs). Among the 6 nodes, 1 (16.67%) were hub node and 3 (50.00%) were ranked in the top 5% of 433 nodes in the network for betweenness centrality. By searching genes in pathways, we found that these two modules participated in several important biological pathways involved in MI, including the toll-like receptor signalling pathway, the MAPK signalling pathway, the PI3K-Akt signalling pathway and apoptosis.

### A potential pathway model of miR-21-5p, the miR-29 family and SP1 in cardiac fibrosis, apoptosis and angiogenesis

**Fig 4:**
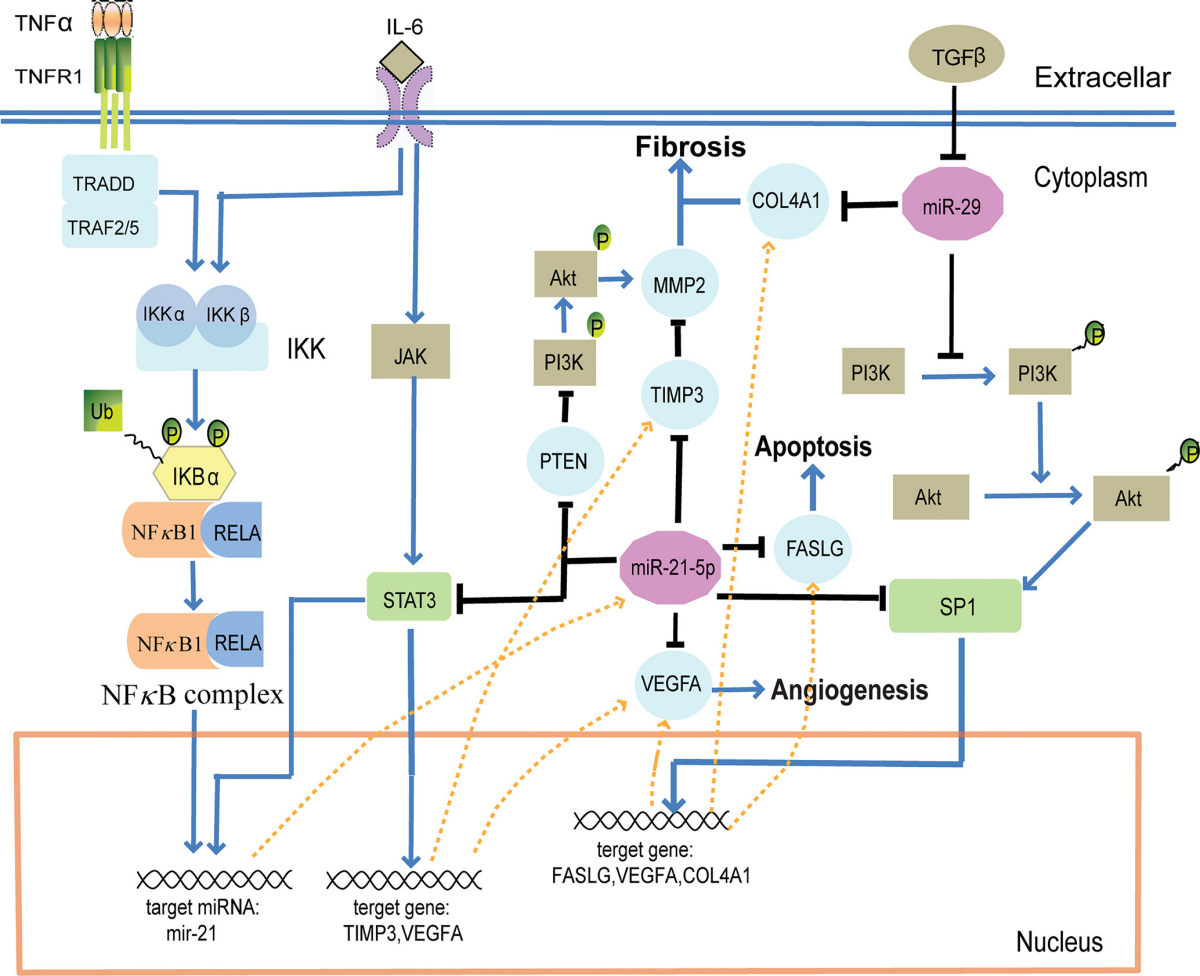
Model of co-regulation of miR-21-5p, the miR-29 family and SP1 involving a biological pathway and the regulatory network. After MI, the expression of TNF-α, IL-6 and TGF-β increased and activated several biological pathways, including JAK and PI3K/AKT pathways. These signal transductions activated several TFs, such as NFκB, STAT3 and SP1, to promote the transcription of miRNAs (e.g. mir-21) and genes (e.g. *TIMP3*, *VEGFA*, *FASLG* and *COL4A1*). miR-21-5p, the miR-29 family and SP1 co-regulated the process of cardiac fibrosis, apoptosis and angiogenesis through several cascades.

To explore the potential co-regulatory mechanisms between miRNAs and TFs in MI, a pathway model demonstrating the co-regulation of miR-21-5p, the miR-29 family (miR-29a-3p, miR-29b-3p and miR-29c-3p) and SP1 in cardiac fibrosis, apoptosis and angiogenesis was proposed (Fig 4). This pathway model was constructed based on the constructed MI-specific miRNA and TF mediated regulatory network and a comprehensive literature review (S3 Fig and S6 Table).

The expression of TNF-α and IL-6 was shown to increase following MI [47–49]. By interacting with their respective receptors, TNF-α triggered IκB (inhibitor of κB) ubiquitination and activated NFκB complex [50], while IL-6 activated STAT3 [51] and IKK (IκB kinase), leading to the up-regulation of miR-21-5p [52]. In the miRNA and TF mediated regulatory network, STAT3, TIMP3, FASLG, SP1 and VEGFA, all targets of miR-21-5p, formed FFLs, and most of the regulatory relationships in these FFLs were validated in previous studies (S3 Fig and S6 Table). STAT3 and miR-21-5p formed a feedback loop that could induce the expression of TIMP3 and VEGFA [49, 51]. Additionally, the up-regulation of miR-21-5p indirectly increased the expression of MMP2 through modulating PTEN [53, 54] and TIMP3 [55], promoting cardiac fibrosis, and decreased the expression of FASLG [56] and VEGFA [55], inhibiting apoptosis and angiogenesis. On the other hand, FASLG and VEGFA were targets of SP1 [57, 58], and miR-21-5p inhibited the expression of SP1 [59] to reduce FASLG and VEGFA activation, thus inhibiting apoptosis and angiogenesis.

The expression of TGF-β was also shown to increase following MI [48], inhibiting the expression of the miR-29 family, which are known to target multiple collagens involved in fibrosis [46]. Indeed, miR-29b-3p has been shown to regulate cardiac fibrosis by modulating COL1A1 either directly or indirectly through SP1 [60], thus creating a miRNA-FFL composed of miR-29b-3p, SP1 and COL1A1. In the miRNA and TF mediated regulatory network, each of the three miR-29 family members formed miRNA-FFLs with COL4A1 and SP1 (S3 Fig), and previous studies have confirmed that COL4A1 is a target of the miR-29 family and that SP1 could regulate the expression of COL4A1 [61–64]. Thus, miR-29 family members, SP1 and multiple collagens were assumed to form FFLs to regulate fibrosis. In addition, according to this pathway module, the miR-29 family might participate in the process of apoptosis and angiogenesis through indirectly modulating the expression of FASLG and VEGFA.

## Discussion

In this study, the first miRNA and TF mediated regulatory network specifically for MI was constructed by merging three types of FFLs: TF-FFL, miRNA-FFL and composite FFL. The results of the randomization test demonstrated that this network was significantly enriched in MI, and several published studies supported the reliability of the network. Additionally, critical regulators and regulatory modules of the network were identified, and a potential pathway model highlighting the co-regulation of miR-21-5p, the miR-29 family and SP1 was proposed, which may provide new clues for deciphering the regulatory mechanisms of MI.

By analyzing the topological properties and modules of the miRNA and TF mediated regulatory network, 6 TFs (JUN, MYC, CEBPA, ETS1, AHR and RELA) were identified as novel and important MI-related TFs, and the cardiac involvement of each was confirmed by recently published studies. Specifically, JUN was reportedly involved in miR-21-mediated injury on cardiac myocytes [65], and was upregulated by reactive oxygen species during MI [66]. MYC interacted with other molecules to enhance cardiovascular cell lineage differentiation and improve functional recovery following acute MI [42]. CEBPA was reported to mediate epicardial activation during heart development and injury [67], while ETS1 was found to interact with SP1 to regulate Fas ligand transcription, an event that could lead to plaque rupture, precipitating MI and sudden death [57]. In patients with acute coronary syndromes (including acute MI and unstable angina pectoris), AHR expression levels were significantly increased compared with those in the stable angina pectoris and control groups [68], and activity of the AHR signal transduction pathway was strongly linked with a reduction of infarct size [69]. Recent studies also reported that MI rats had higher levels of NFκB p65 (RELA, also known as p65) activity in the paraventricular nucleus when compared to sham surgery rats [70]. Collectively, these results suggested the reliability and effectiveness of the constructed network.

Enrichment analysis of biological pathways using 237 MIgenes in the network revealed all the four pathways in cardiovascular diseases pathways: hypertrophic cardiomyopathy (adjusted *p*-value = 4.57×10^−4^), dilated cardiomyopathy (adjusted *p*-value = 8.81×10^−4^), arrhythmogenic right ventricular cardiomyopathy (adjusted *p*-value = 0.0247), and viral myocarditis (adjusted *p*-value = 0.0452). Notably, some pathways related to cancer were also significantly enriched, demonstrating that the dysregulation of these cancer pathways might also lead to MI. These results have therefore also revealed potentially novel relationships between cancer and MI. As is already understood, tumor metastasis to the heart with tumor embolization or direct tumor compression on the coronary arteries may lead to MI [71]. Additionally, significantly-enriched biological pathways for 854 MIgenes we initially selected were examined by implementing the same procedure. As a result, 43 significantly-enriched pathways were identified (S7 Table). However, only 3 pathways in cardiovascular diseases pathways were significantly enriched: hypertrophic cardiomyopathy (adjusted *p*-value = 0.0028), dilated cardiomyopathy (adjusted *p*-value = 0.0060) and viral myocarditis (adjusted *p*-value = 0.0393).

The miRNA and TF mediated regulatory network was constructed based on both experimentally verified and computationally predicted data. To reduce the effect of false positives, data was selected by integrating multiple data sources. Genes and miRNAs related to MI were collected from multiple commonly used data sources, and human TFs were collected from several experimentally verified databases. The regulatory relationships among genes, miRNAs and TFs were extracted from both experimentally verified and computationally predicted sources under stringent analysis standards and parameters. However, we noted that most MI-related genes and miRNAs used in this work have not been confirmed to be causal; the regulatory relationships among genes, miRNAs and TFs were neither complete nor unbiased. Particularly in the pathway model we proposed, although single aspects of the network were supported by primary literatures (S6 Table), these single findings did not necessarily provide evidence to support the entire networks presented as a whole, and further experimental confirmation was warranted. With an improvement of the quantity and quality of these data, the MI-specific miRNA and TF mediated regulatory network will be more accurate and comprehensive. For future studies, the inclusion of more MI-related biological data, such as expression profiles and functional information including GO and/or pathway data, should be considered to improve the informational content of the network.

In summary, the analysis of this miRNA and TF mediated regulatory network identified some critical regulators and regulatory modules for MI. This network could potentially serve as an effective tool for further deciphering the pathogenesis of MI at the transcriptional and post-transcriptional levels.

## Supporting Information

S1 Fig Three types of feed forward loops (FFLs). (TIF)

S2 Fig Cumulative distributions of functional semantic scores of gene pairs in the module and randomly selected MIgene pairs for GO three sub-ontologies. (A) Biological process (BP), p-value = 3.26×10–10. (B) Molecular function (MF), p-value = 1.11×10–6. (C) Cellular component (CC), p-value = 2.25×10–6. The p-value was calculated by the Kolmogorov-Smirnov test.
(TIF)

S3 Fig FFLs in the pathway model and the MI-specific miRNA and TF mediated regulatory network. (TIF)

S1 Table Three types of FFLs based on MI-related data. (XLS)

S2 Table MI-specific miRNA and TF mediated regulatory network. (XLS)

S3 Table MiRNAs and TFs with the highest (top 5%) betweenness centrality in the MI-specific miRNA and TF mediated regulatory network. (DOC)

S4 Table Hub genes, hub miRNAs and hub TFs in the MI-specific miRNA and TF mediated regulatory network. (DOC)

S5 Table Significantly-enriched KEGG pathways for 237 MIgenes in the MI-specific miRNA and TF mediated regulatory network. (DOC)

S6 Table Literature evidence for the regulatory relationships in the pathway model (Fig 4). (DOC)

S7 Table Significantly-enriched KEGG pathways for 854 MIgenes we initially selected. (DOC)
