# Supplementary figures and images for: MicroRNA and Transcription Factor Mediated Regulatory Network Analysis Reveals Critical Regulators and Regulatory Modules in Myocardial Infarction
Source: PLoS One. 2015 Aug 10;10(8):e0135339. doi: 10.1371/journal.pone.0135339 (PMC4530868; doi:10.1371/journal.pone.0135339)

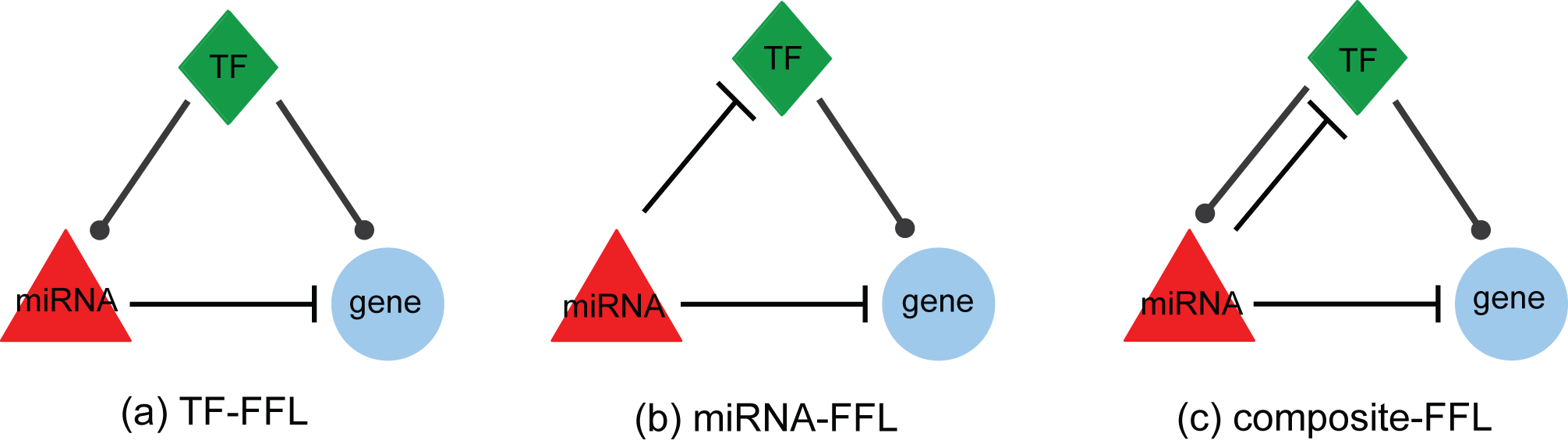

Supplement: S1 Fig — (TIF) [file pone.0135339.s001.tif]

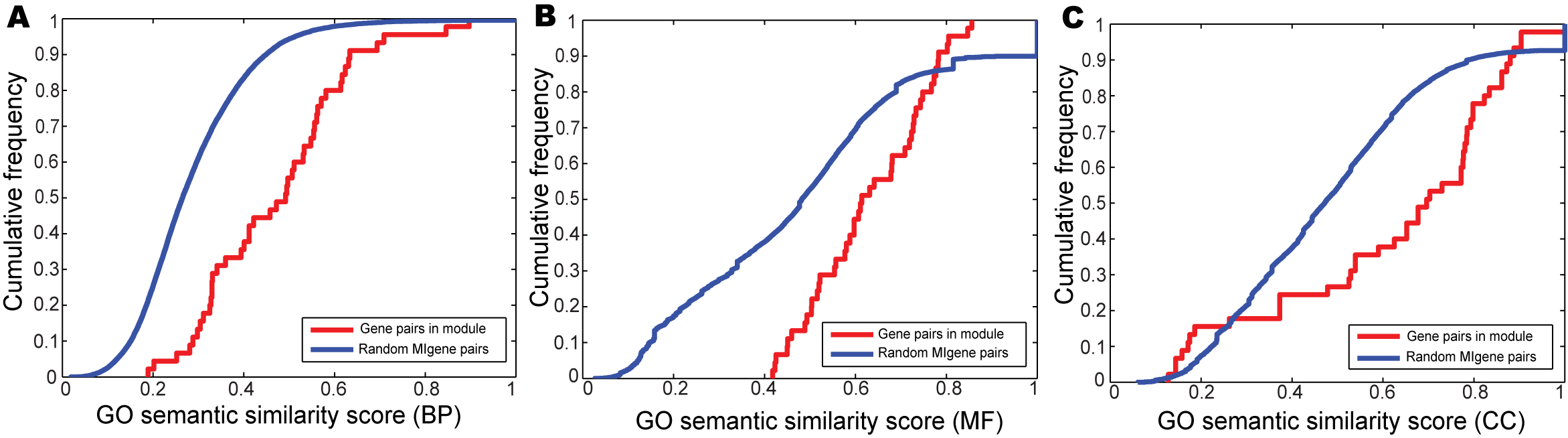

Supplement: S2 Fig — (A) Biological process (BP), p-value = 3.26×10^–10^. (B) Molecular function (MF), p-value = 1.11×10^–6^. (C) Cellular component (CC), p-value = 2.25×10^–6^. The p-value was calculated by the Kolmogorov-Smirnov test. (TIF) [file pone.0135339.s002.tif]

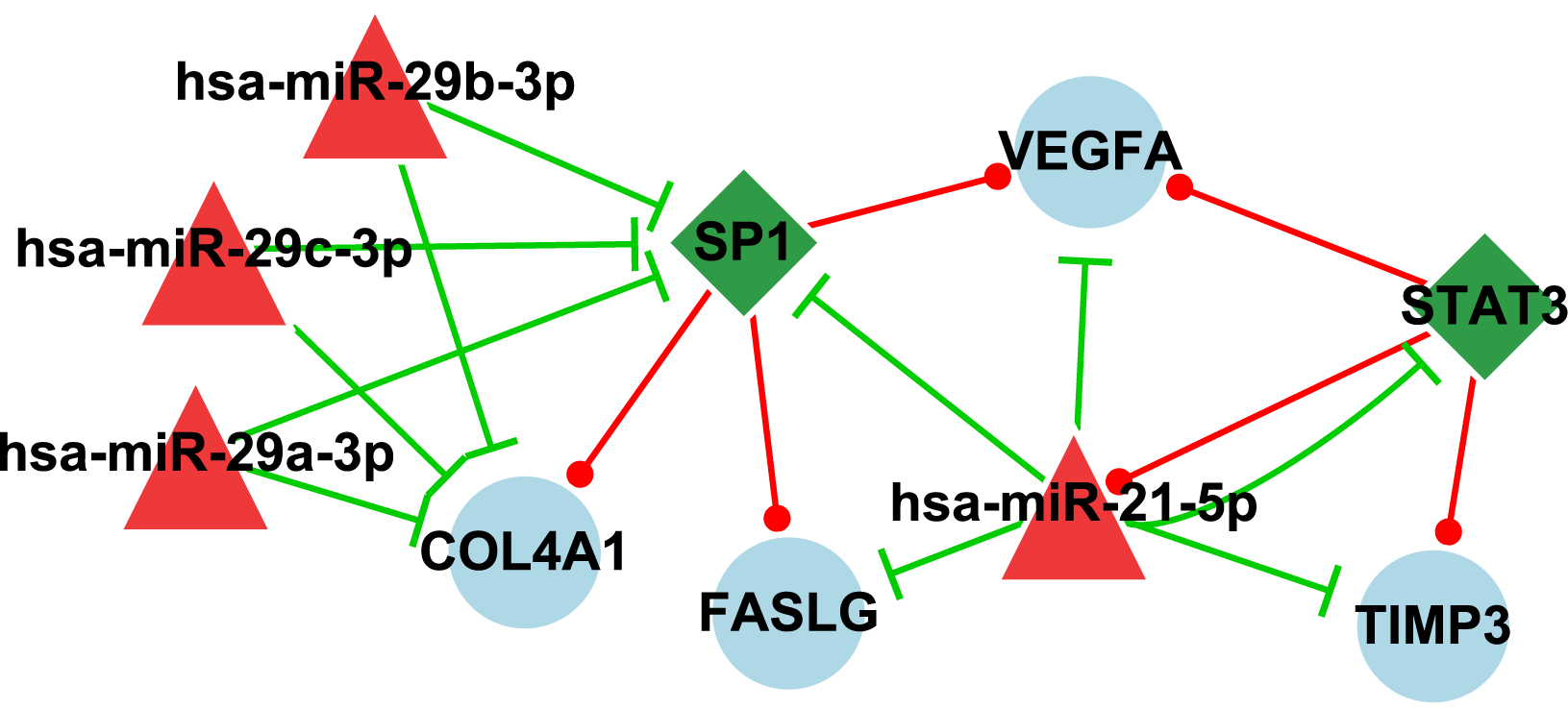

Supplement: S3 Fig — (TIF) [file pone.0135339.s003.tif]
