# Supplementary material for: MicroRNA and Transcription Factor Mediated Regulatory Network Analysis Reveals Critical Regulators and Regulatory Modules in Myocardial Infarction
Source: PLoS One. 2015 Aug 10;10(8):e0135339. doi: 10.1371/journal.pone.0135339 (PMC4530868; doi:10.1371/journal.pone.0135339)
Supplement: S3 Table — (DOC) [file pone.0135339.s006.doc]

**S3 Table.** MiRNAs and TFs with the highest (top 5%) betweenness centrality in the MI-specific miRNA and TF mediated regulatory network.

| **miRNAs** | **betweenness centrality** | **TFs** | **betweenness centrality** |
| --- | --- | --- | --- |
| hsa-miR-21-5p | 9817.24 | ESR1 | 9853.67 |
| hsa-miR-155-5p | 6883.54 | SP1 | 8045.79 |
| hsa-miR-92a-3p | 5506.49 | NFKB1 | 6166.12 |
|  |  | TP53 | 4106.56 |
|  |  | MYC | 3989.11 |
|  |  | STAT3 | 2902.10 |
|  |  | FOXO3 | 2828.92 |
