# Supplementary material for: MicroRNA and Transcription Factor Mediated Regulatory Network Analysis Reveals Critical Regulators and Regulatory Modules in Myocardial Infarction
Source: PLoS One. 2015 Aug 10;10(8):e0135339. doi: 10.1371/journal.pone.0135339 (PMC4530868; doi:10.1371/journal.pone.0135339)
Supplement: S4 Table — (DOC) [file pone.0135339.s007.doc]

## S4 Table. Hub genes, hub miRNAs and hub TFs in the MI-specific miRNA and TF mediated regulatory network.

| **Genes** | **Degree** | **miRNAs** | **Degree** | **TFs** | **Degree** |
| --- | --- | --- | --- | --- | --- |
| CDKN1A | 31 | hsa-miR-155-5p | 88 | SP1 | 102 |
| VEGFA | 30 | hsa-let-7b-5p | 71 | JUN | 54 |
| IGF1 | 24 | hsa-miR-92a-3p | 63 | MYC | 46 |
| PSG1 | 21 | hsa-miR-93-5p | 54 | NFKB1 | 43 |
| TNF | 20 | hsa-miR-21-5p | 53 | ESR1 | 39 |
|  |  | hsa-miR-29b-3p | 39 | NR3C1 | 36 |
|  |  | hsa-miR-29a-3p | 38 | CREB1 | 34 |
|  |  |  |  | CEBPA | 32 |
|  |  |  |  | ETS1 | 32 |
