# Supplementary material for: MicroRNA and Transcription Factor Mediated Regulatory Network Analysis Reveals Critical Regulators and Regulatory Modules in Myocardial Infarction
Source: PLoS One. 2015 Aug 10;10(8):e0135339. doi: 10.1371/journal.pone.0135339 (PMC4530868; doi:10.1371/journal.pone.0135339)
Supplement: S5 Table — (DOC) [file pone.0135339.s008.doc]

## S5 Table. Significantly-enriched KEGG pathways for 237 MIgenes in the MI-specific miRNA and TF mediated regulatory network.

| **Rank** | **KEGG pathways** | ***Adjusted *p*-value** |
| --- | --- | --- |
| 1 | Pathways in cancer | 5.42E-10 |
| 2 | ECM-receptor interaction | 4.68E-07 |
| 3 | Focal adhesion | 4.93E-07 |
| 4 | Small cell lung cancer | 1.08E-04 |
| 5 | Cytokine-cytokine receptor interaction | 1.29E-04 |
| 6 | ^△^Hypertrophic cardiomyopathy (HCM) | 4.57E-04 |
| 7 | ^△^Dilated cardiomyopathy | 8.81E-04 |
| 8 | Bladder cancer | 9.75E-04 |
| 9 | Chronic myeloid leukemia | 0.0025 |
| 10 | Colorectal cancer | 0.0052 |
| 11 | Complement and coagulation cascades | 0.0056 |
| 12 | TGF-beta signaling pathway | 0.0063 |
| 13 | Graft-versus-host disease | 0.0168 |
| 14 | Toll-like receptor signaling pathway | 0.0173 |
| 15 | Hematopoietic cell lineage | 0.0185 |
| 16 | Type I diabetes mellitus | 0.0202 |
| 17 | Neurotrophin signaling pathway | 0.0207 |
| 18 | MAPK signaling pathway | 0.0235 |
| 19 | ^△^Arrhythmogenic right ventricular cardiomyopathy (ARVC) | 0.0247 |
| 20 | Intestinal immune network for IgA production | 0.0353 |
| 21 | Adipocytokine signaling pathway | 0.0391 |
| 22 | Allograft rejection | 0.0396 |
| 23 | p53 signaling pathway | 0.0405 |
| 24 | ErbB signaling pathway | 0.0424 |
| 25 | Apoptosis | 0.0424 |
| 26 | Amyotrophic lateral sclerosis (ALS) | 0.0431 |
| 27 | Pancreatic cancer | 0.0437 |
| 28 | T cell receptor signaling pathway | 0.0447 |
| 29 | Prostate cancer | 0.0448 |
| 30 | ^△^Viral myocarditis | 0.0452 |

Note: ‘^△^’ denotes the pathways belonged to cardiovascular disease pathway in KEGG. ‘*****’ indicates *p*-values adjusted by Benjamini-Hochberg multiple testing correction.
