# Supplementary material for: MicroRNA and Transcription Factor Mediated Regulatory Network Analysis Reveals Critical Regulators and Regulatory Modules in Myocardial Infarction
Source: PLoS One. 2015 Aug 10;10(8):e0135339. doi: 10.1371/journal.pone.0135339 (PMC4530868; doi:10.1371/journal.pone.0135339)
Supplement: S6 Table — (DOC) [file pone.0135339.s009.doc]

**S6 Table.** Literature evidence for the regulatory relationships in the pathway model (Fig. 4).

| **Molecule 1** | **Molecule 1** | **Regulation type** | **Literature validation** | **PubMed ID** |
| --- | --- | --- | --- | --- |
| hsa-miR-21-5p | TIMP3 | a | Yes | 18591254 |
| hsa-miR-21-5p | STAT3 | b | Yes | 18591254 |
| hsa-miR-21-5p | SP1 | b | Yes | 22034194 |
| hsa-miR-21-5p | FASLG | a | Yes | 22038740 |
| hsa-miR-21-5p | VEGFA | a | Yes | 18591254 |
| hsa-miR-21-5p | PTEN | a | Yes | 20560046 |
| hsa-miR-29a-3p | COL4A1 | a | Yes | 20067797 |
| hsa-miR-29a-3p | SP1 | b | No | - |
| hsa-miR-29b-3p | COL4A1 | a | Yes | 20657750 |
| hsa-miR-29b-3p | SP1 | b | Yes | 22297492 |
| hsa-miR-29c-3p | COL4A1 | a | Yes | 18390668 |
| hsa-miR-29c-3p | SP1 | b | No | - |
| SP1 | FASLG | c | Yes | 11970950 |
| SP1 | VEGFA | c | Yes | 19420388 |
| SP1 | COL4A1 | c | Yes | 8422402 |
| STAT3 | TIMP3 | c | Yes | 8898888 |
| STAT3 | hsa-miR-21-5p | d | Yes | 20546595 |
| STAT3 | VEGFA | c | Yes | 19096025 |
| NFκB1 | hsa-miR-21-5p | d | Yes | 20546595 |
| RELA | hsa-miR-21-5p | d | Yes | 20546595 |

Note: ‘a’ : miRNA repression of gene expression

‘b’ : miRNA repression of TF expression

‘c’ : TF regulation of gene expression.

‘d’ : TF regulation of miRNA expression
