# Supplementary material for: MicroRNA and Transcription Factor Mediated Regulatory Network Analysis Reveals Critical Regulators and Regulatory Modules in Myocardial Infarction
Source: PLoS One. 2015 Aug 10;10(8):e0135339. doi: 10.1371/journal.pone.0135339 (PMC4530868; doi:10.1371/journal.pone.0135339)
Supplement: S7 Table — (DOC) [file pone.0135339.s010.doc]

## S7 Table. Significantly-enriched KEGG pathways for 854 MIgenes we initially selected.

| **Rank** | **KEGG pathways** | ***Adjusted *p*-value** |
| --- | --- | --- |
| 1 | Complement and coagulation cascades | 4.79E-21 |
| 2 | Cytokine-cytokine receptor interaction | 5.46E-09 |
| 3 | Hematopoietic cell lineage | 1.35E-08 |
| 4 | Arachidonic acid metabolism | 1.25E-06 |
| 5 | ECM-receptor interaction | 2.25E-06 |
| 6 | Pathways in cancer | 7.15E-06 |
| 7 | Focal adhesion | 1.62E-05 |
| 8 | Small cell lung cancer | 2.36E-05 |
| 9 | Type II diabetes mellitus | 0.0021 |
| 10 | Allograft rejection | 0.0021 |
| 11 | ^△^Hypertrophic cardiomyopathy (HCM) | 0.0028 |
| 12 | Linoleic acid metabolism | 0.0031 |
| 13 | PPAR signaling pathway | 0.0035 |
| 14 | T cell receptor signaling pathway | 0.0037 |
| 15 | Toll-like receptor signaling pathway | 0.0038 |
| 16 | Metabolism of xenobiotics by cytochrome P450 | 0.0055 |
| 17 | Type I diabetes mellitus | 0.0057 |
| 18 | ^△^Dilated cardiomyopathy | 0.0060 |
| 19 | NOD-like receptor signaling pathway | 0.0076 |
| 20 | Colorectal cancer | 0.0101 |
| 21 | Jak-STAT signaling pathway | 0.0101 |
| 22 | Asthma | 0.0112 |
| 23 | Pancreatic cancer | 0.0113 |
| 24 | Prion diseases | 0.0115 |
| 25 | Apoptosis | 0.0129 |
| 26 | Bladder cancer | 0.0136 |
| 27 | Prostate cancer | 0.0141 |
| 28 | Intestinal immune network for IgA production | 0.0144 |
| 29 | Chronic myeloid leukemia | 0.0146 |
| 30 | Cell adhesion molecules (CAMs) | 0.0151 |
| 31 | Autoimmune thyroid disease | 0.0193 |
| 32 | Fc epsilon RI signaling pathway | 0.0194 |
| 33 | mTOR signaling pathway | 0.0215 |
| 34 | Adipocytokine signaling pathway | 0.0267 |
| 35 | Aldosterone-regulated sodium reabsorption | 0.0277 |
| 36 | Vascular smooth muscle contraction | 0.0309 |
| 37 | Drug metabolism | 0.0318 |
| 38 | Renal cell carcinoma | 0.0356 |
| 39 | ^△^Viral myocarditis | 0.0393 |
| 40 | Chemokine signaling pathway | 0.0411 |
| 41 | Neurotrophin signaling pathway | 0.0413 |
| 42 | TGF-beta signaling pathway | 0.0435 |
| 43 | Graft-versus-host disease | 0.0488 |

Note: ‘^△^’ denotes the pathways belonged to cardiovascular disease pathway in KEGG. ‘*****’ indicates *p*-values adjusted by Benjamini-Hochberg multiple testing correction.
